# Non-transplant patients with haematologic malignancies have better survival outcome than recipients of haematopoietic stem cell transplant (HSCT) after admission to intensive care unit (ICU)

**DOI:** 10.1186/2197-425X-3-S1-A243

**Published:** 2015-10-01

**Authors:** Y Corcia Palomo, T Knight Asorey, I Espigado Tocino, L Martín Villén, A Gutiérrez Pizarrayo, J Garnacho Montero

**Affiliations:** Intensive Care Service, H Virgen del Rocio, Sevilla, Spain; Haematology and Haemotherapy Service, H Virgen del Rocio, Sevilla, Spain; Red Española de Investigación en Patología Infecciosa REIPI, H Virgen del Rocio, Sevilla, Spain

## Introduction

Patients with haematological malignancies admitted to ICU have a high mortality rate. Identifying predictors of mortality and survival may be useful for clinical decision making and can help to improve survival. The differences in mortality rate and other outcomes between recipients of HSCT and non-transplant haematologic patients admitted to the ICU have been poorly studied.

## Objectives

To compare survival rate after intensive care admission between recipients of HSCT and non-transplant patients with haematologic malignancies.

## Patients and Methods

Prospective observational study of all consecutive patients with haematologic malignancies admitted to the ICU. Patients had been undergone to an HSCT or not. Period: December 2012 through April 2014. Variables that were analyzed included: demographics haematological disease, stage of the haematological disease, main reason for admission to the ICU, severity of illness (APACHE II y SOFA), organ failure, need of organ support therapy, death rate in the Unit, in hospital and in the following 28 days after admission. The Chi square test was used to analyse categorical variables and the U Mann -Whitney test to compare quantitative variables.

## Results

63 consecutive patients were included and their data analyzed. Twenty-seven (42.9%) have undergone an HSCT and 36 did not. Acute leukemias were the main hematological diagnosis that determined the transplant (11 patients, 40.7%) and 51.9% of transplanted patients were admitted in ICU in complete remission. HSCT recipients tended to be younger [median age 51 (34-55) vs. 57 (43-66); p = 0.01] and they were admitted to the ICU in a worst clinical condition SOFA [10 (8-14) vs. 8 (4-11); p = 0.01] and APACHE II [26 (19-29) vs. 21 (16-28); p = 0.32]. The most frequent cause of admission to the ICU was respiratory failure (77.8 %), without differences between both groups (p = 0.54). However, recipients of HSCT needed more frequently invasive mechanical ventilation (85.2% vs. 52.8%, p = 0.007) and suffered significantly more liver failure (77.8% vs. 30.6%; p < 0.001) and renal failure (85.2% vs. 44.4%, p < 0.001). Haemodynamic failure was also more frequent in the HSCT recipients (88.9% vs. 63.9%; p = 0.02). Hospital mortality rate 28 days after ICU admission of patients with HSCT was higher than for non-HSCT patients (92.6% vs. 44.4%; p < 0.001). There were no significant differences between both groups in the rest of the analyzed variables.

## Conclusions

Recipients of an HSCT have a significantly higher mortality rate and worse survival than non-transplant haematologic patients after intensive care admission. The causes for such poor outcome could be the worst clinical condition at admission, more need for invasive mechanical ventilation and higher rate of organ failure in HSCT recipients. Knowing the reasons for these differences merit further investigation and may help to improve survival of both patients settings.

